# Is there room for combined modality treatments? Dosimetric comparison of boost strategies for advanced head and neck and prostate cancer

**DOI:** 10.1093/jrr/rrt067

**Published:** 2013-07

**Authors:** Joanna Góra, Johannes Hopfgartner, Peter Kuess, Brigita Paskeviciute, Dietmar Georg

**Affiliations:** 1 Department of Radiooncology, Division of Medical Radiation Physics, Comprehensive Cancer Center, Medical University Vienna/AKH Wien Waehringer Guertel 18–20, 1090 Vienna, Austria; 2 EBG MedAustron GmbH, Marie Curie-Straße 5, A-2700 Wiener Neustadt, Austria; 3 Christian Doppler Laboratory for Medical Radiation Research for Radiation Oncology, Medical University Vienna, Austria

**Keywords:** VMAT, IMIT, IMPT, combined treatment, boost, H&N, prostate cancer

## Abstract

The purpose of the study was to determine the dosimetric difference between three emerging treatment modalities—volumetric-modulated arc therapy (VMAT), intensity-modulated proton beam therapy (IMPT) and intensity-modulated carbon ion beam therapy (IMIT)—for two tumour sites where selective boosting of the tumour is applied. For 10 patients with locally advanced head and neck (H&N) cancer and 10 with high-risk prostate cancer (PC) a VMAT plan was generated for PTV_initial_ that included lymph node regions_,_ delivering 50 Gy (IsoE) for H&N and 50.4 Gy (IsoE) for PC patients. Furthermore, separate boost plans (VMAT, IMPT and IMIT) were created to boost PTV_boost_ up to 70 Gy (IsoE) and 78 Gy (IsoE) for H&N and PC cases, respectively. Doses to brainstem, myelon, larynx and parotid glands were assessed for H&N cases. Additionally, various OARs (e.g. cochlea, middle ear, masticator space) were evaluated that are currently discussed with respect to quality of life after treatment. For PC cases, bladder, rectum and femoral heads were considered as OARs. For both tumour sites target goals were easily met. Looking at OAR sparing, generally VMAT + VMAT was worst. VMAT + IMIT had the potential to spare some structures in very close target vicinity (such as cochlea, middle ear, masticator space ) significantly better than VMAT + IMPT. Mean doses for rectal and bladder wall were on average 4 Gy (IsoE) and 1.5 Gy (IsoE) higher, respectively, compared to photons plus particles scenarios. Similar results were found for parotid glands and larynx. Concerning target coverage, no significant differences were observed between the three treatment concepts. Clear dosimetric benefits were observed for particle beam therapy as boost modality. However, the clinical benefit of combined modality treatments remains to be demonstrated.

## INTRODUCTION

It has been shown that dose escalation in the tumour volume can significantly increase local control [1, 2]. For radiotherapy of high-risk cancer, treating volumes of potential risk to certain dose levels and simultaneous or subsequent boosting of the visible tumour became standard of care. In this context various boost strategies ranging from brachytherapy over external photon to particle beam therapy options have been explored [3–6]. Another (radiobiological) ‘degree of freedom’ in designing optimal treatments can be utilized when considering hypo- or hyper-fractionation. Finally, the challenge is to determine which strategy is most applicable when considering efficiency, costs, convenience, time and impact on quality of life.

The rapidly evolving field of particle therapy (protons as well as carbon ions), and the increasing number of facilities worldwide make this modality more commonly available and hence easier to incorporate into a boost treatment regimen. Nevertheless, photon beam therapy does not stand still and the recent development of rotational intensity-modulated radiation therapy (IMRT) like volumetric-modulated arc therapy (VMAT) is very promising, both with respect to treatment plan quality and treatment efficiency, i.e. highly conformal dose distributions can be achieved within a few minutes [7, 8]. Palma *et al*. compared VMAT with IMRT for 10 prostate cancer (PC) patients and the VMAT technique resulted in more favourable dose distributions and reduced the monitor units required compared with IMRT [9]. Similar conclusions were drawn by Wiehle *et al*. for patients with head and neck (H&N) tumours [10].

The scope of this study was to determine and assess the dosimetric differences between three emerging treatment modalities for two treatment sites where selective boosting of the tumour is applied, i.e. advanced H&N and prostate cancer (PC). More specifically, VMAT, intensity-modulated proton beam therapy (IMPT) and intensity-modulated carbon ion beam therapy (IMIT) were explored for these scenarios. Furthermore, their combinations were evaluated to answer the question if there is room for combined modality treatment.

## MATERIALS AND METHODS

### Patient data and image acquisition

This retrospective study was performed for 10 patients with locally advanced H&N cancer and 10 patients with high-risk PC. All of them underwent IMRT treatment at the Medical University of Vienna/AKH Vienna.

Patients with H&N tumours were immobilized with the double-layered HeadSTEP^™^ mask combined with the BiteSTEP^™^ system (Elekta, Crawley, UK). For prostate cases the ProSTEP^™^ system (Elekta, Crawley, UK) was utilized for external immobilization of the legs, while simultaneously an endorectal balloon (filled with 40 cm^3^ air) was used for pushing the posterior part of the rectal wall out of the high dose region. For treatment planning, images were acquired with a multi-slice CT scanner (Siemens, Erlangen, Germany). In general, 4-mm slice thickness was used but treatment site-specific scanning protocols were applied.

### Volume definition and dose prescription

#### H&N cases

On individual planning CTs, two clinical target volumes (CTVs) were delineated. The CTV_initial_ consisted of the visible tumour itself, affected lymph nodes and additional areas of potential risk while CTV_boost_ comprised only the tumour. Following our clinical practice both CTVs were expanded by a uniform 5-mm margin in order to create the planning target volumes (PTV_initial_ and PTV_boost_). A dose of 50 Gy (IsoE) was prescribed to the PTV_initial_ and an additional 20 Gy (IsoE) was prescribed to the PTV_boost_. It should be emphasized that all doses in this manuscript are expressed as isoeffective doses, which are the absorbed doses that, if delivered under the reference treatment conditions, would produce the same effects on a given biological system as the dose delivered under the actual treatment conditions, with all other treatment conditions being identical. This is in accordance with the most recent recommendation of the ICRU and the IAEA in order to make doses delivered by photon as well as particle modalities comparable [11].

Furthermore, the following organs at risk (OARs) were contoured, for which our in-house-developed constraints were applied to drive treatment plan optimization (see Table 1): brainstem, myelon, larynx and both parotid glands. Furthermore, a body structure was generated, consisting of the patients' outer contour minus the PTV.

**Table 1.** In-house applied OAR constraints

H&N IMRT protocol				PC IMRT protocol		
Myelon	Brainstem	Contralateral parotid gland	Larynx	Rectum	Bladder	Femoral heads
D_max_< 48 Gy (IsoE)	D_max_< 50 Gy (IsoE)	D_mean_< 26 Gy (IsoE)	D_mean_< 45 Gy (IsoE)	V _50 Gy (IsoE)_ < 50%	V _30 Gy (IsoE)_ < 80%	V _50 Gy (IsoE)_ < 5% D_max_ < 60 Gy (IsoE)
				V _60 Gy (IsoE)_ < 45%	V _50 Gy (IsoE)_ < 50%	
				V _65 Gy (IsoE)_ < 40%	V _55 Gy (IsoE)_ < 45%	
				V _70 Gy (IsoE)_ < 20%	V _70 Gy (IsoE)_ < 20%	
				D_max_ < 110%	D_max_ < 110%	

V_*x* Gy (IsoE)_ = volume receiving *x* Gy (IsoE), D_max_ = maximal dose, D_mean_ = mean dose to the organ.

Additionally, we were interested in doses delivered to other structures, which are normally not accounted for, but still have an influence on the patient's quality of life [12, 13]. These additional organs were both cochleae, lower and upper lip, mandible (anterior and posterior part), maxilla (anterior and posterior), both middle ears, occipital scalp, larynx (separated into glottic and super glottic part), oral cavity, cerebellum, pharyngeal constrictor (inferior, superior and middle), base of tongue, both submandibular salivary glands, mucosa of lips and cheeks, masticator space (ipsi- and contralateral), both temporomandibular joints, soft palate and oesophagus.

#### PC cases

Similar to the H&N group, two CTVs were defined. The CTV_initial_ was defined as the prostate gland including seminal vesicles and involved lymph nodes, and the CTV_boost_ comprised the prostate gland exclusively. The following three PTVs were created according to the department treatment protocol for high-risk PC patients. Firstly, the PTV_initial_ was generated by isotropic 10-mm expansion of the CTV_initial_. The second PTV (PTV_10mm_) was created of the CTV_boost_ plus an additional safety margin of 10 mm in all directions. Finally, the PTV_boost_ was defined as a uniform extension of the CTV_boost_ by 5 mm. The intended dose to the PTV_initial_ was 50.4 Gy (IsoE), with a simultaneously integrated dose boost of 56 Gy (IsoE) administered to the PTV_10mm_. An additional dose of 22 Gy (IsoE) was prescribed to the PTV_boost_ with a sequential boost.

Regarding OARs, bladder, rectum and femoral heads were considered. The rectal wall was derived from the rectum structure by extracting 3 mm from the external wall of the rectum. In a similar manner the bladder wall was defined. Furthermore, to simulate the bladder without contrast agent, the electron density of the opacified bladder was virtually changed to 1 (relative to water). The rectal balloon (originally filled with air) was modified to be water equivalent, in order to avoid range uncertainties related to heterogeneity changes in the path of the particles and to mimic clinical particle beam therapy scenarios. OAR constraints for plan optimization are given in Table 1. The body structure was generated similarly as for H&N cases.

### Treatment planning strategies

For each patient an initial VMAT plan was generated to deliver the prescribed dose to the large PTV_initial_ that included lymph node regions, i.e. of 50 Gy (IsoE) for H&N cases and 50.4/56 Gy (IsoE) to the PTV_initial_/PTV_10mm_ for PC cases. Thereafter, three separate boost plans were created (VMAT, IMPT and IMIT) to deliver the additional dose to the PTV_boost_. Treatment plans were considered clinically acceptable if 95% of the respective target volume was covered with at least 95% of the prescribed dose and simultaneously all OAR constraints were fulfilled [14].

#### VMAT plans

For the VMAT technique, the treatment planning system (TPS) Monaco (V.3.2; Elekta, St Louis, USA) was used. The first dose level for the large PTV_initial_ was delivered within two arcs (2 × 360°). Due to the much simpler geometry of the PTV_boost_, a single arc (360°) was found to be sufficient for the boost plans. For all VMAT plans 10-MV photon beams were used. The maximum number of control points was 150 while the dose rate was defined as 150 MU/min with a minimum segment width of 0.5 cm. The dose was calculated using a Monte Carlo (MC) algorithm with a standard deviation of 3% on a grid size of 3 × 3 × 3 mm^3^.

#### Proton (IMPT) plans

Proton boost plans were generated with the TPS XiO (V4.41; CMS/Elekta, Crawley, UK). In general two beam ports were used. While for H&N cases the incident beam angles were 45°/90° or 315°/270°, depending on the respective tumour location, in the prostate group two lateral opposed beams (90° and 270°) were applied. Dose calculation was performed with a pencil-beam algorithm on the grid size 3 × 3 × 3 mm^3^. The energy-dependent spot sigma was calculated internally by the planning software based on an initial spot sigma of 3 mm in air. The lateral spot spacing of the pencil beams was 5 mm. The energy-dependent distance between two spot layers was designed to be in the order of a single Bragg peak (width at 80% of the peak maximum) at the respective depth. A global RBE of 1.1 (relative high energy photons) was assumed.

#### Carbon ion (IMIT) plans

For carbon ion boost plans the TPS TRiP98 (V1001c, GSI, Darmstadt, Germany) was used. This software was especially developed for optimization and calculation of carbon ion dose distributions, both physical and biological ones. The software was designed to be compatible with 3D active dose-shaping devices like a raster scan system [15]. The physical dose is calculated by a fast pencil beam algorithm, without consideration of multiple Coulomb scattering for low-scattering carbon ions. The biological dose is determined by multiplying the physical dose with an RBE value in each voxel, which is calculated according to the local effect model (LEM1) model [16, 17]. For this purpose an α/β ratio of 2 Gy was applied for targets as well as normal tissue for both patient categories, while for the rectum and bladder 4 Gy and 5 Gy were used, respectively [18–21].

Beam arrangements were the same as for IMPT. In order to ensure comparable proton and carbon ion delivery scenarios, similar beam spot-defining parameters were chosen. The sigma value for the beam spots was 3.4 mm, while the distance between two successive layers was set to 3 mm. The lateral spot spacing of the pencil beams was 5 mm. Dose computation was carried out on the CT grid of 1 × 1 × 4 mm^3^. For evaluation purposes it was afterwards recalculated to a 3 × 3 × 3 mm^3^ grid.

### Treatment plan evaluation

With an in-house adapted version of the software platform CERR (V4.1) dose matrices of the initial VMAT plans and the boost plans were mapped together in order to sum up the doses on a voxel-by-voxel basis. In other words, evaluation of the final results was performed by creating dose-volume histograms (DVHs) for the whole treatment, independently from the TPS. Hence, as an outcome for each patient, three DVHs were generated consisting of summed doses from VMAT plus the VMAT boost plan (VMAT + VMAT), the VMAT plus the IMPT boost plan (VMAT + IMPT) and the VMAT plus the IMIT boost plan (VMAT + IMIT). The dosimetric differences between the three different treatment approaches were evaluated. Average DVHs were created over the entire patient populations (H&N, PC).

For each of the composite treatment plans the following dose indices regarding target volumes were reported: median dose (D_median_), dose to 95% of the volume (D_95%_), volume covered by 95% of the prescribed dose (V_95%_), homogeneity index (HI) as well as conformity number (CN). HI was defined as the ratio of the difference between the D_2%_ and D_98%_ and the median dose. CN was calculated as a ratio of the squared target volume covered by 95% isodose and the total target volume multiplied by the volume covered by the 95% isodose. More detailed information regarding these indices can be found in the literature [14, 22, 23]. Concerning serial OARs (e.g. brainstem and myelon), average doses (D_mean_) as well as near maximum doses (D_2%_) were investigated. For parallel OARs (e.g. rectal and bladder wall) other DVH parameters (e.g. V_30 Gy (IsoE)_, V_60 Gy (IsoE)_, D_30%_, D_90%_) were derived additionally.

### Statistical evaluation

In total 60 plans were analysed. The data were assumed to follow a normal distribution and a paired *t*-test was applied for each dosimetric parameter for comparing all possible combinations, i.e. PLAN_VMAT+VMAT_ vs PLAN_VMAT+IMPT_; PLAN_VMAT+VMAT_ vs PLAN_VMAT+IMIT_, and PLAN_VMAT+IMPT_ vs PLAN_VMAT+IMIT_. Due to the large number of required tests and to minimize the probability of false positive results, a Bonferroni adjustment was applied. *P*-values for every possible treatment pair were multiplied by 3 before concluding statistical significance of the result (*P* < 0.05). The software SPSS Statistics (V17.0, SPSS Inc., Chicago, USA) was utilized for this purpose [24, 25].

## RESULTS

**Fig. 1.**
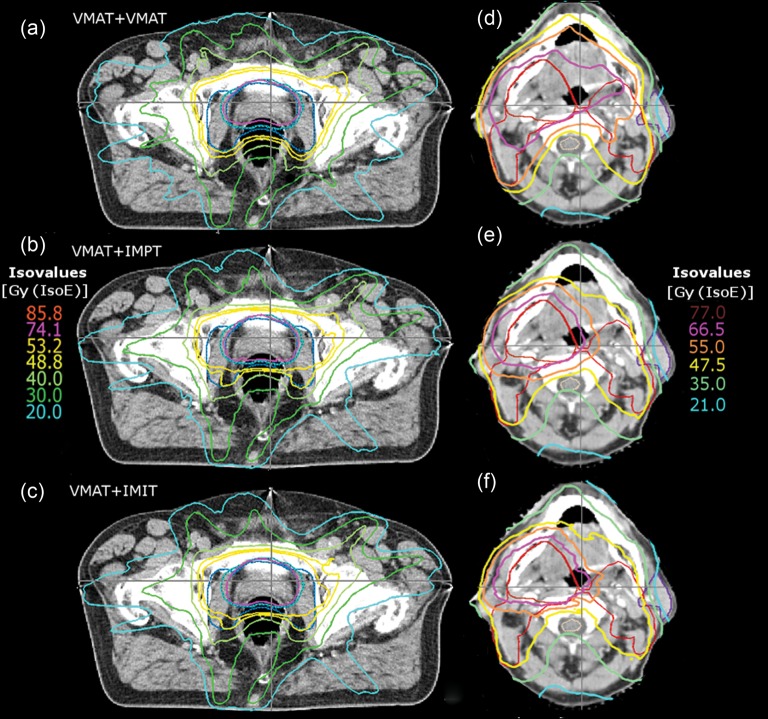
Representative isodose distributions resulting from combined treatment modalities. Left column: PC patient, right column: H&N patient: (**a** and **d**) VMAT + VMAT. (**b** and **e**) VMAT + IMPT. (**c** and **f**) VMAT + IMIT.

Each of the created plans fulfilled the given target constraints. Figure 1 represents the typical dose distribution for PC cases (left) and H&N cancer cases (right). Similarly, doses to all OARs were within the limits, except for the larynx in nine H&N cases, as the structure was inside the PTV, and for the femoral heads in some PC cases (*n* = 3) where averaged doses exceeded the constraints.

### Dosimetric summary of H&N cases

**Fig. 2.**
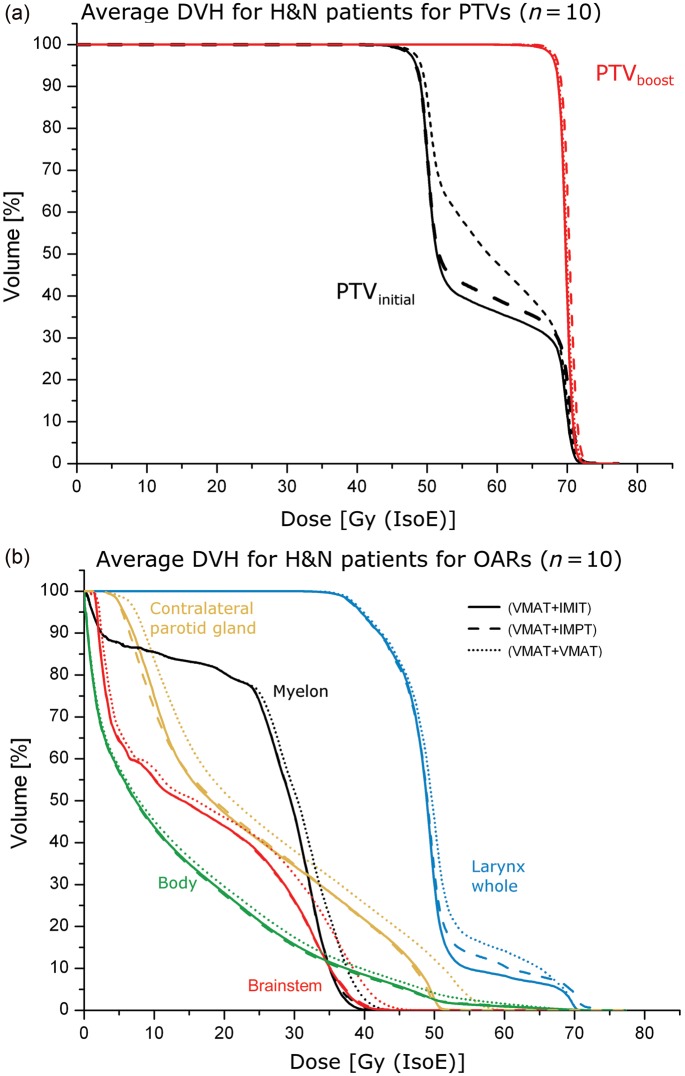
Averaged DVHs over the entire H&N patient population. (**a**) DVH of the target volumes. (**b**) DVH of OARs.

Figure 2 exemplifies the patient population averaged DVHs. The specific dosimetric values regarding targets and OARs are addressed individually in the following paragraphs.

#### Target coverage

Table 2 summarizes the results for the different treatment modalities. Concerning the initial planning target volumes, D_median_ was on average up to 10% higher for the VMAT + VMAT approach, compared to the others. Therefore, systematically lower values of HI were observed for this treatment option. On the other hand, DVHs of the respective initial PTVs and CTVs revealed that differences between the particle modalities were marginal.

**Table 2.** Summary of target dose indices for H&N patients

		Modalities			*P*-values		
Organ	Index	VMAT + VMAT	VMAT + IMPT	VMAT + IMIT	VMAT + VMAT vs VMAT + IMPT	VMAT + VMAT vs VMAT + IMIT	VMAT + IMPT vs VMAT + IMIT
PTV_initial_	D_median_ [Gy (IsoE)]	58.2 (4.5)	53.8 (3.9)	53.4 (1.3)	<0.05	<0.05	NS
	D_95%_ [Gy (IsoE)]	49.2 (0.7)	48.4 (0.5)	48.3 (0.5)	<0.05	<0.05	NS
	V_95%_[%]	98.6 (0.9)	97.6 (1.2)	97.8 (1.0)	<0.05	<0.05	NS
	HI	39.4 (4.6)	45.3 (4.1)	45.1 (2.4)	<0.05	<0.05	NS
	CN	0.54 (0.0)	0.61 (0.0)	0.62 (0.0)	<0.05	<0.05	<0.05
CTV_initial_	D_median_ [Gy (IsoE)]	61.1 (5.8)	56.9 (7.7)	58.6 (6.1)	<0.05	<0.05	NS
	D_95%_ [Gy (IsoE)]	49.9 (0.6)	49.2 (0.3)	49.4 (0.2)	<0.05	NS	NS
	V_95%_[%]	99.6 (0.5)	99.5 (0.5)	99.5 (1.0)	<0.05	<0.05	NS
	HI	36.2 (4.5)	40.9 (5.5)	40.9 (4.4)	<0.05	<0.05	NS
PTV_boost_	D_median_ [Gy (IsoE)]	70.0 (0.4)	70. 4 (0.3)	69.4 (0.3)	<0.05	NS	<0.05
	D_95%_ [Gy (IsoE)]	68.4 (0.3)	68.7 (0.5)	68.6 (0.4)	NS	NS	<0.05
	V_95%_[%]	99.4 (0.7)	99.6 (0.4)	99.5 (0.8)	NS	<0.05	<0.05
	HI	5.7 (1.8)	5.4 (1.6)	5.5 (1.6)	NS	NS	NS
	CN	0.62 (0.1)	0.70 (0.1)	0.78 (0.0)	<0.05	<0.05	<0.05
CTV_boost_	D_median_ [Gy (IsoE)]	70.1 (0.4)	70.5 (0.4)	69.4 (0.4)	<0.05	NS	<0.05
	D_95%_ [Gy (IsoE)]	68.9 (0.2)	69.2 (0.4)	69.0 (0.3)	<0.05	NS	<0.05
	V_95%_ [%]	99.9 (0.0)	100 (0.0)	100 (0.0)	NS	NS	NS
	HI	4.0 (1.2)	4.3 (0.7)	4.3 (1.4)	NS	NS	NS

V_95%_ = volume receiving 95 % of the prescribed dose, D_median_ = median dose, D_95%_ = dose delivered to 95 % of the volume, HI = homogeneity index, CN = conformity number, in brackets () = standard deviation, NS = result statistically not significant.

D_95%_ and V_95%_ for the PTV_initial_ and the CTV_initial_ were found to be significantly different in favour of sole photon treatments compared to combined photon and particle therapy modalities, although being only higher by up to 2.5%. For the PTV_boost_ and CTV_boost_ no significant difference could be obtained for those indices. Near minimum as well as maximum doses were very similar with deviations of less than 1% for all modalities. Due to the specific physical characteristics of particles, combined modality treatments (VMAT + IMPT, and VMAT + IMIT) resulted in significantly higher CN than VMAT + VMAT.

#### Sparing of primary OAR

Table 3 gives an overview of resulting OAR doses. For all considered OARs, D_mean_ and D_2%_ for the VMAT + VMAT treatment were found to be significantly worse compared with the other methods. Moreover, the VMAT + IMIT modality spared the myelon, brainstem and the larynx the most. By adopting particle beams for the boost, maximum doses to the myelon as well as to the brainstem could be constrained on average below 37 Gy (IsoE), compared to up to 40 Gy (IsoE) for sole photon treatment.

**Table 3.** Summary of OARs dose indices for H&N patients

		Modalities			*P*-values		
Organ	Index	VMAT + VMAT	VMAT + IMPT	VMAT + IMIT	VMAT + VMAT vs VMAT + IMPT	VMAT + VMAT vs VMAT + IMIT	VMAT + IMPT vs VMAT + IMIT
Parotid gland	D_mean_ [Gy (IsoE)]	25.9 (8.6)	23.1 (7.7)	23.0 (8.0)	<0.05	<0.05	NS
	D_2%_ [Gy (IsoE)]	53.6 (2.8)	49.2 (0.8)	49.3 (1.3)	<0.05	<0.05	NS
Myelon	D_mean_ [Gy (IsoE)]	26.9 (4.0)	25.7 (3.9)	25.6 (3.9)	<0.05	<0.05	NS
	D_2%_ [Gy (IsoE)]	39.5 (2.8)	37.5 (2.7)	36.6 (2.7)	<0.05	<0.05	<0.05
Brainstem	D_mean_ [Gy (IsoE)]	18.5 (9.6)	17.2 (8.5)	16.9 (8.6)	NS	<0.05	NS
	D_2%_ [Gy (IsoE)]	39.6 (8.1)	37.0 (9.6)	36.6 (6.5)	<0.05	<0.05	NS
Larynx (whole)	D_mean_ [Gy (IsoE)]	51.4 (6.7)	50.5 (5.8)	49.7 (4.6)	<0.05	NS	NS
	D_2%_ [Gy (IsoE)]	58.2 (8.1)	58.2 (9.6)	57.3 (8.6)	NS	NS	NS
Body	D_mean_ [Gy (IsoE)]	14.6 (3.5)	13.6 (3.3)	13.5 (3.3)	<0.05	<0.05	<0.05
	D_2%_ [Gy (IsoE)]	56.8 (2.6)	51.7 (2.4)	50.7 (1.7)	<0.05	<0.05	<0.05
	V_5 Gy (IsoE)_ [%]	58.0 (14.4)	56.8 (14.5)	56.8 (14.5)	<0.05	<0.05	NS
	V_35 Gy (IsoE)_ [%]	12.9 (3.5)	11.5 (2.9)	11.1 (3.0)	<0.05	<0.05	NS

V_*x* Gy (IsoE)_ = volume receiving *x* Gy (IsoE), D_mean_ = mean dose, D_2%_ = dose delivered to 2% of the volume, HI = homogeneity index, CN = conformity number, in brackets () = standard deviation, NS = result statistically not significant.

Looking at D_mean_, D_2%_, V_5 Gy (IsoE)_ and V_35 Gy (IsoE)_ of the body structure, a significant increase in the dose was observed for the VMAT + VMAT method compared to VMAT + IMPT and VMAT + IMIT (on average 1 Gy (IsoE) for all the indices, but for D_2%_ it was 6 Gy (IsoE) higher). Furthermore, concerning D_mean_ and D_2%_ VMAT + IMIT was superior over VMAT + IMPT.

#### Sparing of the additional OARs

Concerning the sparing of additional OARs, which might have an impact on the quality of life after treatment, the VMAT + VMAT method appeared to be the worst in most of the cases. For almost all OARs considered, D_mean_ and D_2%_ were significantly higher_,_ with mean dose differences mostly around 3 Gy (IsoE). Additionally, for some of the structures like ipsilateral cochlea, ipsilateral middle ear, base of tongue, upper lip etc., VMAT + IMIT showed significant benefits even over VMAT + IMPT. The detailed results are summarized in Table 4.

**Table 4.** Summary of additional OARs (associated with quality of life after radiation) dose indices for H&N patients

	D_mean_ [Gy (IsoE)]			*P*-values			D_2%_ [Gy (IsoE)]			*P*-values		
	VMAT + VMAT	VMAT + IMPT	VMAT + IMIT	VMAT + VMAT vs VMAT + IMPT	VMAT + VMAT vs VMAT + IMIT	VMAT + IMPT vs VMAT + IMIT	VMAT + VMAT	VMAT + IMPT	VMAT + IMIT	VMAT + VMAT vs VMAT + IMPT	VMAT + VMAT vs VMAT + IMIT	VMAT + IMPT vs VMAT + IMIT
Occipital scalp	18.2	15.7	15.7	<0.05	<0.05	NS	39.3	34.2	34.3	<0.05	<0.05	NS
Cerebellum	22.1	19.2	19.3	<0.05	<0.05	NS	45.5	38.8	38.9	<0.05	<0.05	NS
Cochlea ips.	28.6	25.0	23.1	<0.05	<0.05	<0.05	37.97	33.6	31.0	<0.05	<0.05	NS
Cochlea contr.	22.4	20.7	20.4	<0.05	<0.05	NS	27.1	25.1	25.1	<0.05	<0.05	NS
Middle ear ips.	32.4	28.2	26.1	<0.05	<0.05	<0.05	49.9	45.5	42.6	<0.05	<0.05	<0.05
Middle ear contr.	22.3	20.1	20.1	<0.05	<0.05	NS	31.6	29.2	29.4	NS	<0.05	NS
Maxilla ant.	31.0	24.4	23.9	<0.05	<0.05	<0.05	42.1	35.8	41.0	<0.05	NS	NS
Maxilla post.	46.5	39.4	38.9	<0.05	<0.05	NS	66.3	66.0	64.8	NS	NS	NS
Mandible ant.	43.0	34.6	34.1	<0.05	<0.05	NS	51.4	42.9	41.6	<0.05	<0.05	NS
Mandible post.	48.6	40.8	40.3	<0.05	<0.05	NS	60.1	54.8	54.2	NS	NS	NS
Mandible whole	52.8	47.4	49.5	<0.05	NS	NS	70.3	70.7	70.2	NS	NS	NS
Inferior pharyngeal constrictor	51.6	50.9	50.1	<0.05	NS	NS	56.8	57.4	56.4	NS	NS	NS
Middle pharyngeal constrictor	62.6	60.9	60.5	<0.05	NS	NS	70.1	69.8	69.1	NS	NS	NS
Superior pharyngeal constrictor	67.9	65.9	65.0	<0.05	<0.05	<0.05	71.4	71.8	71.1	NS	NS	<0.05
Base of tongue	67.7	64.5	63.2	<0.05	<0.05	<0.05	70.9	71.3	70.8	NS	NS	<0.05
Glottic larynx	50.8	50.1	49.0	<0.05	NS	NS	54.9	55.1	54.2	NS	NS	<0.05
Supraglottic larynx	57.1	55.9	55.1	NS	<0.05	<0.05	66.1	65.0	64.2	NS	NS	NS
Oesophagus	32.6	32.3	32.1	NS	NS	NS	48.5	48.5	47.1	NS	NS	NS
Parotis ipsilateral	55.4	53.5	53.0	NS	<0.05	NS	70.5	70.8	70.2	NS	NS	<0.05
Submand. salivary gland ips.	64.8	64.2	63.7	NS	NS	NS	70.9	71.2	70.7	NS	NS	NS
Submand. salivary gland contr.	59.8	53.9	52.0	<0.05	<0.05	NS	65.9	60.4	57.3	<0.05	<0.05	NS
Mucosa of lips and cheeks	45.8	39.7	39.4	<0.05	<0.05	NS	68.8	67.7	67.5	NS	NS	NS
Upper lip	22.8	18.8	18.4	<0.05	<0.05	<0.05	36.3	32.6	31.4	<0.05	<0.05	<0.05
Lower lip	34.2	28.2	27.9	<0.05	<0.05	NS	45.9	40.2	34.4	<0.05	<0.05	<0.05
Oral cavity	62.5	57.1	55.7	<0.05	<0.05	<0.05	70.9	70.7	69.7	NS	NS	<0.05
Soft palate	64.7	63.5	62.4	NS	<0.05	<0.05	70.9	71.1	63.0	NS	<0.05	<0.05
Masticator space ips.	53.7	54.7	52.9	NS	NS	<0.05	71.1	71.6	70.8	<0.05	NS	<0.05
Masticator space contr.	36.6	29.3	29.6	<0.05	<0.05	NS	64.5	59.4	57.4	<0.05	<0.05	NS
Temporomandibular joint ips.	37.6	36.4	34.4	NS	<0.05	NS	52.5	51.0	49.6	NS	NS	NS
Temporomandibular joint contr.	15.9	13.7	13.7	<0.05	<0.05	NS	24.6	22.6	22.3	NS	<0.05	NS

D_mean_ = mean dose, D_2%_= dose delivered to 2% of the volume, HI = homogeneity index, CN = conformity number, NS = result statistically not significant.

### Dosimetric summary for prostate cases

**Fig. 3.**
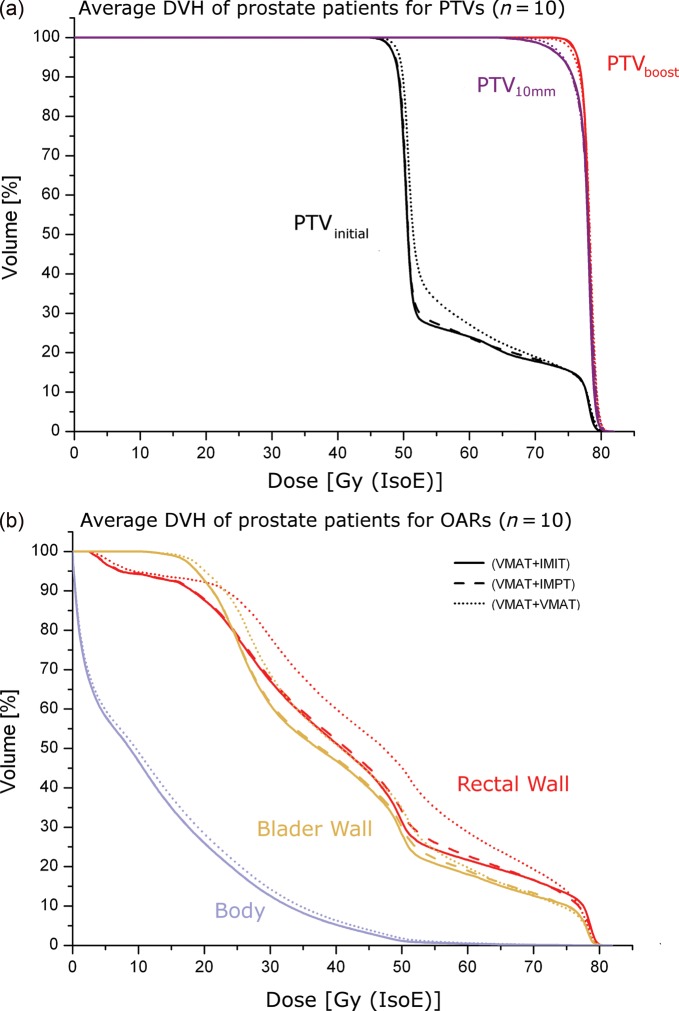
Averaged DVHs over the entire prostate patient population. (**a**) DVH of the target volumes. (**b**) DVH of OARs.

Figure 3 illustrates the patient population averaged DVHs of the target volumes as well as the OARs.

#### Target coverage

In Table 5 the final outcome of the planning comparison is summarized. For PC cases similar results as for H&N cases were generally observed regarding target indices, except for D_median_. The respective dosimetric difference was not that pronounced (within 2%) and hence considered to be not clinically relevant. This was caused by the much larger volumetric difference between the PTV_initial_ and the PTV_boost_ between H&N and PC cases. Statistically significant differences between sole photon treatments and combined photon and particle beam treatments were observed for the PTV_initial_, where for D_95%_ differences were up to 0.8 Gy (IsoE) and for V_95%_ up to 1.5%. Again, for the PTV_boost_, the CTV_boost_ and the PTV_10 mm_ no significant differences were observed for those indices.

**Table 5.** Summary of target dose indices for PC patients

		Modalities			*P*-values		
Organ	Index	VMAT+ VMAT	VMAT+ IMPT	VMAT+ IMIT	VMAT + VMAT vs VMAT + IMPT	VMAT + VMAT vs VMAT + IMIT	VMAT + IMPT vs VMAT + IMIT
PTV_initial_	D_median_ [Gy (IsoE)]	52.3 (3.2)	51.1 (1.7)	51.2 (1.4)	NS	NS	NS
	D_95%_ [Gy (IsoE)]	49.4 (0.5)	48.7 (0.4)	48.6 (0.4)	<0.05	<0.05	NS
	V_95%_ [%]	99.1 (0.6)	97.7 (0.8)	97.6 (0.8)	<0.05	<0.05	NS
	HI [%]	58.2 (4.3)	60.8 (2.4)	60.7 (2.6)	<0.05	<0.05	NS
	CN	0.62 (0.0)	0.68 (0.0)	0.68 (0.0)	<0.05	<0.05	NS
CTV_initial_	D_median_ [Gy (IsoE)]	53.1 (4.8)	52.0 (4.2)	52.4 (4.0)	<0.05	NS	NS
	D_95%_ [Gy (IsoE)]	50.0 (0.3)	49.6 (0.2)	49.4 (0.3)	<0.05	<0.05	<0.05
	V_95%_ [%]	99.9 (0.1)	99.9 (0.1)	99.9 (0.1)	NS	NS	NS
	HI [%]	55.5 (4.7)	57.2 (4.1)	56.7 (4.0)	<0.05	NS	NS
PTV_10 mm_	D_median_ [Gy (IsoE)]	78.2 (0.4)	78.1 (0.4)	77.9 (0.3)	NS	NS	NS
	D_95%_ [Gy (IsoE)]	74.1 (0.9)	74.0 (0.9)	74.1 (1.1)	NS	NS	NS
	V_95%_ [%]	100 (0.0)	100 (0.0)	100 (0.0)	NS	NS	NS
	HI [%]	9.1 (1.5)	10.5 (1.8)	10.3 (1.8)	NS	NS	NS
	CN	0.26 (0.0)	0.35 (0.0)	0.37 (0.0)	<0.05	<0.05	<0.05
PTV_boost_	D_median_ [Gy (IsoE)]	78.3 (0.5)	78.2 (0.4)	78.1 (0.3)	NS	NS	NS
	D_95%_ [Gy (IsoE)]	76.0 (0.7)	76.3 (0.6)	76.4 (0.5)	NS	NS	NS
	V_95%_ [%]	98.8 (1.0)	99.6 (0.5)	99.7 (0.3)	NS	NS	NS
	HI [%]	6.0 (1.7)	4.9 (1.0)	4.7 (0.9)	NS	<0.05	NS
	CN	0.78 (0.1)	0.81 (0.1)	0.83 (0.1)	<0.05	<0.05	NS
CTV_boost_	D_median_ [Gy (IsoE)]	78.5 (0.5)	78.3 (0.5)	78.1 (0.3)	NS	NS	NS
	D_95%_ [Gy (IsoE)]	77.6 (0.4)	77.5 (0.4)	77.4 (0.4)	NS	NS	NS
	V_95%_ [%]	100 (0.0)	100 (0.0)	100 (0.0)	NS	NS	NS
	HI [%]	2.8 (0.5)	2.8 (0.4)	2.9 (0.4)	NS	NS	NS

V_95%_ = volume receiving 95 % of the prescribed dose, D_median_ = median dose, D_95%_ = dose delivered to 95 % of the volume, HI = homogeneity index. CN = conformity number, in brackets () = standard deviation, NS = result statistically not significant.

Variations of minimum as well as maximum doses to target volumes were found to be within 1% for all scenarios. On the other hand, on average systematically lower HI values were obtained for sole photon treatments (up to 2.6% for the PTV_initial_), except for the PTV_boost_ and the CTV_boost_. Moreover, combined treatments (VMAT + IMPT, and VMAT + IMIT) resulted in significantly higher CN.

#### OAR sparing

The dosimetric overview of resulting bladder and rectal wall doses for the three modalities is presented in Table 6.

**Table 6.** Summary of OARs dose indices for PC patients

		Modalities			*P*-values		
Organ	Index	VMAT + VMAT	VMAT + IMPT	VMAT + IMIT	VMAT + VMAT vs VMAT + IMPT	VMAT + VMAT vs VMAT + IMIT	VMAT + IMPT vs VMAT + IMIT
Rectal wall	D_mean_ [Gy (IsoE)]	47.2 (2.8)	43.0 (2.6)	42.6 (2.8)	<0.05	<0.05	<0.05
	D_2%_ [Gy (IsoE)]	78.7 (0.6)	79.0 (0.6)	79.1 (0.5)	NS	<0.05	NS
	V_30 Gy (IsoE)_ [%]	78.2 (6.2)	67.8 (7.8)	67.2 (7.9)	<0.05	<0.05	<0.05
	V_60 Gy (IsoE)_ [%]	28.7 (5.5)	22.7 (3.3)	21.7 (4.8)	<0.05	<0.05	NS
	D_30%_ [Gy (IsoE)]	59.2 (4.5)	52.3 (2.7)	51.4 (3.0)	<0.05	<0.05	<0.05
	D_90%_ [Gy (IsoE)]	22.4 (6.6)	18.4 (5.9)	18.2 (5.9)	<0.05	<0.05	<0.05
Bladder wall	D_mean_ [Gy (IsoE)]	43.5 (3.9)	41.7 (3.6)	41.4 (3.6)	<0.05	<0.05	NS
	D_2%_ [Gy (IsoE)]	78.6 (0.6)	78.5 (0.6)	78.4 (0.5)	NS	NS	NS
	V_30 Gy (IsoE)_ [%]	68.9 (11.9)	61.6 (10.6)	61.1 (10.6)	<0.05	<0.05	<0.05
	V_60 Gy (IsoE)_ [%]	19.7 (7.6)	18.9 (6.7)	19.0 (6.2)	NS	NS	NS
	D_30%_ [Gy (IsoE)]	52.2 (5.7)	50.0 (5.5)	47.4 (8.3)	<0.05	NS	NS
	D_90%_ [Gy (IsoE)]	23.5 (3.7)	21.6 (3.3)	19.6 (6.1)	<0.05	NS	NS
Body	D_mean_ [Gy (IsoE)]	13.8 (2.9)	12.9 (2.8)	12.9 (2.8)	<0.05	<0.05	NS
	D_2%_ [Gy (IsoE)]	49.0 (2.3)	47.1 (2.1)	47.0 (2.2)	<0.05	<0.05	NS
	V_5 Gy (IsoE)_ [%]	59.7 (12.3)	58.1 (12.0)	58.0 (12.1)	<0.05	<0.05	NS
	V_39 Gy (IsoE)_ [%]	6.9 (1.9)	5.7 (1.7)	5.8 (1.7)	<0.05	<0.05	NS

V_x Gy (IsoE)_ = volume receiving *x*_Gy (IsoE)_, D_mean_ = mean dose, D_x%_ = dose delivered to *x*% of the volume, HI = homogeneity index, CN = conformity number, in brackets () = standard deviation, NS = result statistically not significant.

No significant differences were observed for D_2%_ between the methods. In general, regarding all other dose and dose-volume indices (D_mean_, V_30 Gy (IsoE)_, V_60 Gy (IsoE),_ D_30%_, D_90%_) VMAT + VMAT plans were significantly worse compared to photons combined with particles, except for those for the bladder wall (V_60 Gy (IsoE)_, D_30%_, D_90%_). Moreover, for some indices of the rectal wall (V_30 Gy (IsoE)_, D_30%_, D_90%_) and for V_30 Gy (IsoE)_ of the bladder wall, combining photons with carbon ions spared the organs significantly better than VMAT + IMPT.

Regarding D_mean_, D_2%_, V_5 Gy (IsoE)_ and V_39 Gy (IsoE)_ of the body structure, a significant increase in the dose was observed for the VMAT + VMAT method compared with the other techniques, for example for D_mean_ the difference was almost 7% and for D_2%_ over 4%.

The femoral heads were spared best with VMAT + IMPT (results not shown). D_mean_, D_2%_, and V_35 Gy (IsoE)_ was found to be the worst for VMAT + IMIT, while the values of V_40 Gy (IsoE)_, V_50 Gy (IsoE)_ and V_60 Gy (IsoE)_ were highest for combined VMAT + VMAT plans. However, almost none of those differences reached statistical significance.

## DISCUSSION

In the case of dose-escalated treatments in the H&N region clear differences between sole photon treatment and treatments combining photons and particle beam therapy were observed. First, the dosimetric characteristics of particle beam therapy resulted in improved dose conformity. While for the boost target no dosimetric differences were observed, reduced doses outside the boost target could be achieved. That feature of combined treatment techniques can be seen in Fig. 1 as well as in the steeper DVH slope for the PTV_initial_ in Figs 2a and 3a.

Furthermore, for OARs VMAT + IMPT as well as VMAT + IMIT showed systematic benefits over VMAT + VMAT. For H&N cases, on average a 10% reduction of the mean dose to the contralateral parotid gland was obtained for these combined modality treatments. Such dose limitation can cause clinically relevant reduction of xerostomia, which often burdens the patients' quality of life after radiation treatment [26]. Moreover, the investigation of all other ‘additional’ OAR structures showed clear benefits in favour of VMAT + IMPT and VMAT + IMIT. These structures are generally not considered, but Rosenthal *et al.* [12] associated them with the patients' quality of life after radiation treatment. Therefore, we decided to include those structures in our analysis in order to reinforce their findings. It should be highlighted that the structures were not *a priori* included in the optimization procedure but analyzed in the following treatment plan evaluation. For structures which were situated very closely around the targets (e.g. ipsilateral cochlea and middle ear), carbon ions showed advantages over protons due to their much smaller lateral penumbra. In general, there is not much dosimetric data available in recent literature regarding this issue. However, Schwartz *et al.* [13] focused on a long-term swallowing disorder after IMRT. With prescribed doses up to 70 Gy, mean doses of 63 Gy delivered to the supraglottic larynx and the superior pharyngeal constrictor, doses of around 18 Gy to the glottis part and 29 Gy to the ipsilateral parotid gland were found. Based on our clinical experience even sole VMAT treatment achieves similar dose levels to IMRT with enhanced delivery efficiency. Here, our results (see Table 4) did not support that, and higher doses were reported for the above-mentioned structures, except for the supraglottic larynx. The reason could be related to the fact that the larynx as well as both of the parotid glands were fully encompassed by the PTV.

For prostate cases similar characteristics concerning the overall dose distributions were found as for H&N cases. Target volume parameters showed better results for combined modality treatments employing particles, although target median dose differences between the various treatment concepts were not that pronounced. This can be explained by the larger difference of the volumes of the initial and the boost target. Consequently, DVH curves varied more strongly for smaller volumes than for larger volumes, and the overall DVH was dictated by that of the large PTV. Dosimetric differences regarding the rectal as well as the bladder wall were predominantly obtained in the low- and medium dose area, while in the hight dose range no beneficial effect of one or the other boost modality could be observed. Highest dose values to the femoral head were obtained for VMAT + IMIT, which was associated with an overestimated RBE in the entrance channel of the beams by the LEM1 model, as well as the optimization process of the TRiP98 version used. These limitations were counteracted in newer versions of the planning system incorporating the new LEM4 model for biological optimization [27, 28].

A uniform dose-escalation approach to the entire boost volumes was pursued in this study, which represents the current standard. This concept of delivering a homogeneous dose to all areas of proven or potential risk is currently under discussion [1]. Selective boosting of suspicious tumour subvolumes (‘dose painting’) paved the way from physical to biological conformal treatment. The more widespread use and associated research based on advanced functional imaging modalities like positron emission tomography (PET) and magnetic resonance imaging or spectroscopy will help to visualize and localize, those tumour subvolumes that are characterized by poor prognostic factors (e.g. high metabolism, hypoxia and elevated cell proliferation) [29, 30]. This concept of incorporating such information in the treatment-planning process and applying selective dose boosts (dose painting) will require more interdisciplinary research but has large potential to further improve radiotherapy [31]. In this context and when using particle beam therapy, Bassler *et al.* [32] and Lühr *et al.* [33] proposed to consider the LET instead of the dose for boosting subvolumes. This principle and its potential to sterilize areas characterized by high oxygen enhancement ratios (OER) and concomitant radioresistance is a further step towards biologically adapted radiation therapy (ART) [34]).

In this study standard fractionation schedules, i.e. 2 Gy (IsoE) fractions, were assumed in order to allow a direct comparison of resulting dose distributions. Dose effects, originating from different fractionation schemes for tumour and OAR volumes are challenging. The investigation of altered fractionation for the boost volume was beyond the scope of our investigation. However, the potential of hypo-fractionated treatment regimens was promoted especially for PC [35, 36] and large randomized trials are ongoing at the M.D. Anderson Cancer Center [37] and in the RTOG group [38]. The results of these trials will help to draw final conclusions in that respect. On the other hand, altered fractionation schedules for H&N cancer were investigated as well [39].

Another slight limitation of the current study is that not all potentially available boost techniques were considered, since brachytherapy (BT) was not included. The combination of external beam radiotherapy (EBRT) and BT has become a common treatment approach, especially for prostate and gynaecological cancer [40, 41]. The dose delivered by BT is extremely conformal, allowing high doses within the tumour with sharp dose gradients towards surrounding structures [6]. In the context of this study and to limit the overall study design, the main focus was given to EBRT delivery techniques.

In summary, dosimetric differences between multi-modal boosting strategies and their potential impact on the patients' quality of life after treatment were investigated. Sole photon treatments based on rotational IMRT (VMAT + VMAT) performed worse compared to treatment regimens employing a combination of photon and charged particle beam therapy (VMAT + IMPT, and VMAT + IMIT). VMAT + IMIT had the potential to spare some structures in very close target vicinity significantly better than VMAT + IMPT. However, the clinical benefit of combined modality treatments remains to be demonstrated.

## FUNDING

Financial support by The European Community's Seventh Framework Programme (FP7 2007–2013) under Grant Agreement No. 215849-2 (Project PARTNER), and the Federal Ministry of Economy, Family and Youth, and the National Foundation for Research, Technology and Development is gratefully acknowledged.
